# An advanced recording and analysis system for the differentiation of guinea pig cough responses to citric acid and prostaglandin E_2_ in real time

**DOI:** 10.1371/journal.pone.0217366

**Published:** 2019-05-22

**Authors:** Jianguo Zhuang, Lei Zhao, Xiuping Gao, Fadi Xu

**Affiliations:** Pathophysiology Program, Lovelace Respiratory Research Institute, Albuquerque, NM, United States of America; National Yang-Ming University, TAIWAN

## Abstract

Cough number and/or sound have been used to assess cough sensitivity/intensity and to discriminate cough patterns in clinical settings. However, to date, only manual counting of cough number in an offline manner is applied in animal cough studies, which diminishes the efficiency of cough identification and hinders the diagnostic discrimination of cough patterns, especially in animals with pulmonary diseases. This study aims to validate a novel recording/analysis system by which cough numbers are automatically counted and cough patterns are comprehensively differentiated in real time. The experiment was carried out in conscious guinea pigs exposed to aerosolized citric acid (CA, 150 mM) and prostaglandin E_2_ (PGE_2_, 0.43 mM). Animal body posture (video), respiratory flow, and cough acoustics (audio) were simultaneously monitored and recorded. Cough number was counted automatically, and cough sound parameters including waveform, duration, power spectral density, spectrogram, and intensity, were analyzed in real time. Our results showed that CA- and PGE_2_-evoked coughs had the same cough numbers but completely different patterns [individual coughs vs. bout(s) of coughs]. Compared to CA-evoked coughs, PGE_2_-evoked coughs possess a longer latency, higher cough rate (coughs/min), shorter cough sound duration, lower cough sound intensity, and distinct cough waveforms and spectrograms. A few mucus- and wheeze-like coughs were noted in response to CA but not to PGE_2_. In conclusion, our recording/analysis system is capable of automatically counting the cough number and successfully differentiating the cough pattern by using valuable cough sound indexes in real time. Our system enhances the objectivity, accuracy, and efficiency of cough identification and count, improves the intensity evaluation, and offers ability for pattern discrimination compared to traditional types of cough identification. Importantly, this approach is beneficial for assessing the efficacy of putative antitussive drugs in animals without or with pulmonary diseases, particularly in cases without significant change in cough number.

## Introduction

Cough is an important respiratory defense mechanism [1, 2] and one of the most common symptoms in clinical complaints [3]. Both cough number and sound have been clinically used to evaluate cough sensitivity and intensity [4–13]. In addition, the analysis of cough acoustic differences (frequency, duration and amplitude) has been applied to discriminate cough patterns, especially in diagnosis of human pulmonary diseases and guidance of the relevant therapies [12, 14–18]. Although the animal cough model has been widely employed in cough pathophysiology and therapy, the current recording/analysis system applied in the model obviously has an unmet requirement for the sufficient determination of cough severity and discrimination of cough pattern.

A typical cough response in conscious animals can be identified by appearance of the following three key signals: (1) a transient and great change in the respiratory flow; (2) a cough sound; and (3) a cough-related body posture (splaying of the front feet and/or forward stretching of the neck with an opening mouth) [4]. With the current animal cough recording/analysis system, cough has been mainly identified by the respiratory flow change, which is sometimes combined with cough sound; this system does not include comprehensive analysis of cough acoustics [19–26] and is rarely associated with the observation (much less recording) of cough-related body posture [23–26]. There are several specific disadvantages in the current system. First, cough numbers were only counted manually in the system, and such counting is time consuming. Second, use of this system may lead to an inaccuracy of cough count. A cough is a transient event. The cough sound was recorded and analyzed in studies using mice [23], guinea pigs [24, 25, 27], pigs [28, 29], and weaned calves [30] to distinguishing a cough [with the peak of power spectral density (PSD) at ~1.5 kHz)] from a sneeze sound (with PSD peak at 3.5–6.5 kHz) [24, 25, 27, 28]. However, this PSD analysis and subsequent cough count were considered only in an offline manner in which observation of the corresponding body posture change was absent. Thus, the risk of subjective judgement exists due to a mismatch of the three transient signals in real time. Third, acoustic analysis of cough sound, such as the intensity and duration, has been used in humans to reflect cough severity [4–13]. Usually, remarkably increased cough sound intensity and prolonged cough sound duration are correlated to severe airway inflammation in patients [12], but unfortunately, such acoustic analysis is lacking in animal cough analysis. Fourth, the previously used system failed to determine cough patterns that are closely related to disease diagnosis and therapeutic strategies in clinical settings. Cough sound waveforms (phases) and spectrograms usually have been examined to discriminate cough pattern. For example, coughs with mucus were distinguished by unique existence of vertical lines in the spectrograms, while those with wheezes were characterized by a series of horizontal bands [31–33]. The vertical lines in these studies are represented by transient lack of spectrogram signals at some time points. In other words, the spectrogram signals are intermittent over the cough duration. The horizontal bands mean that the intensity of cough sound frequencies is distributed in several layers but not even. This analysis has been employed to interpret distinct coughs in patients with idiopathic pulmonary fibrosis, chronic obstructive pulmonary disease, asthma or laryngitis [14, 31–35], but these analyses have not been applied in animal models. Collectively, the recording/analysis system currently utilized in animal cough models does not meet simultaneous monitoring, recording, and analysis of the three key signals mentioned above in real time. This lack diminishes the objectivity, accuracy, and efficiency of cough identification and count. The goal of this study was to develop a novel animal cough recording/analysis system that would minimize the above disadvantages and to validate this system by the comprehensive differentiation of the cough responses to inhalation of citric acid (CA) or prostaglandin E_2_ (PGE_2_). CA inhalation provokes individual loud coughs (Type I), while PGE_2_ inhalation induces bout(s) of smaller and quieter coughs (Type II), which have been defined in humans and conscious guinea pigs [36–42]. We used this new system to automatically count cough numbers, determine cough sound intensity and duration, discriminate cough patterns in real time and compare these parameters between the two types of coughs.

Previous studies have shown that CA exposure induces an immediate and remarkable elevation in ventilation in conscious lambs [43], while inhalation of PGE_2_ fails to change ventilation in the majority of the tested dogs (4/6) [44]. It has been well documented that inhalation of aerosolized CA and PGE_2_ provokes Type I and Type II coughs via mainly acting on vagal sensory TRPV1 and EP_3_ receptors respectively [15, 27, 42, 45–49]. These results encourage us to test whether inhalation of aerosolized CA and PGE_2_ would affect the ventilation in guinea pigs and if so, to what extent the evoked ventilatory responses were different.

## Materials and methods

### Animals and their adaptation

The present study was approved by the Institutional Animal Care and Use Committee (IACUC) and Institutional Biosafety Committee of the Lovelace Respiratory Research Institute. All facilities were accredited by the Association for Assessment and Accreditation of Laboratory Animal Care (AAALAC) International. The guidelines for guinea pig housing, environment, and comfort described in the Guide for the Care and Use of Laboratory Animals (7^th^ Edition, National Research Council) were strictly followed.

A total of 21 pathogen-free male Dunkin-Hartley guinea pigs (200–250 g, Charles River Laboratories, Inc. Wilmington, MA) were ordered and quarantined for 14 days before the experiments. The animals had access to food and water ad libitum with temperature and humidity ranging from 22–26°C and 30–65%, respectively.

After quarantine, the animals were individually placed in a whole-body, unrestrained, plethysmograph chamber (model PLY3215, Buxco Electronics Inc., Troy, NY) for ~40 min once a day for three continuous days before the cough test. Immediately after habituation, guinea pigs were weighed and randomly divided into three groups, as described in the experimental protocols section.

### Setup of the recording system

**Fig 1:**
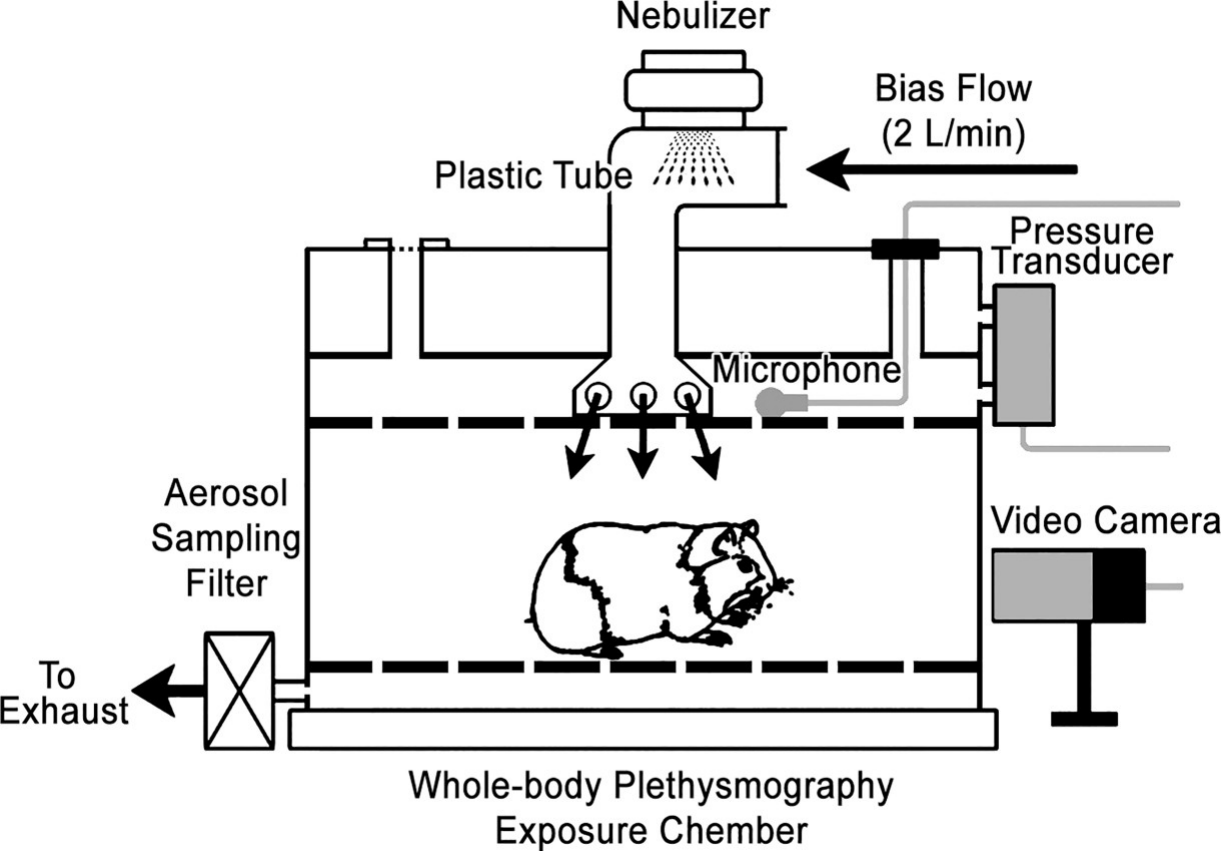
A cartoon showing the exposure chamber and the setup of cough recording system. Arrows point to the flow direction. The signals generated by video camera, microphone, and pressure transducer were collected and digitized continuously through a PowerLab system (ADInstruments Inc.) and recorded into computer files using LabChart 8.0 Pro software (ADInstruments Inc.).

The setup of our recording system is presented in Fig 1. The top of the plethysmograph chamber was connected with a plastic tube attached by a vibrating mesh nebulizer head (Ireland Ltd., Galway, Ireland, AG-AL1100). A normoxic gas mixture (21% O_2_ and 79% N_2_) driven by a nebulizer controller was pushed into the chamber and sucked out by a bias flow regulator with the in- and out-flow volumes balanced. Three lines of signals (video, audio, and respiratory flow) were monitored, recorded, and analyzed. An electret condenser lavalier microphone (model ECM-V1BMP, sensitivity: 43 dB, Sony, Japan) system was mounted in the roof of the chamber to record sound with the manufacturer’s technical parameters to determine the sound intensity. A video camera (Microsoft LifeCam Studio) was placed outside of the chamber to monitor animal body posture. A Buxco pneumotachograph (pressure transducer) was attached to the chamber for recording the respiratory flow. Both the sound and flow signals were collected, digitized at a sampling rate of 20 kHz via a PowerLab unit (model 8/35, ADInstruments Inc., Colorado Springs, CO) and recorded continuously on computer files using a Dell desktop computer (XPS 8930, Dell, Round Rock, Texas) with LabChart 8.0 Pro software (ADInstruments Inc.). Raw signals and analyzed data, including respiratory flow, respiratory rate, sound waveforms, sound spectrogram, cough sound intensity and duration, cough and sneeze counts, were displayed in real-time on a computer monitor (U3417W, Dell Ultrasharp 34 in. curved ultrawide, Dell).

### Aerosol exposure to CA and PGE_2_

The plethysmograph chamber was continuously flushed with normoxic gas mixtures (2 L min^-1^) mingled with or without a given nebulized aqueous solution. Vehicle, CA, or PGE_2_ solution was aerosolized by using the nebulizer. The output rate of delivered aerosol was 0.5 ml min^-1^ with a volume median diameter of 2.5–4.0 μm (per the manufacturer's indications). The plethysmograph chamber was placed in a standard exhaust fume hood (size: 3 x 6 ft).

### Analysis of cough

Cough numbers were both manually and automatically counted in this study, and the relevant data were compared to determine the accuracy of the automatic count. All calculations and analyses mentioned below were performed using the inner functions, extensions, and modules of the LabChart Pro software.

#### Manual counting of cough numbers in real time

**Fig 2:**
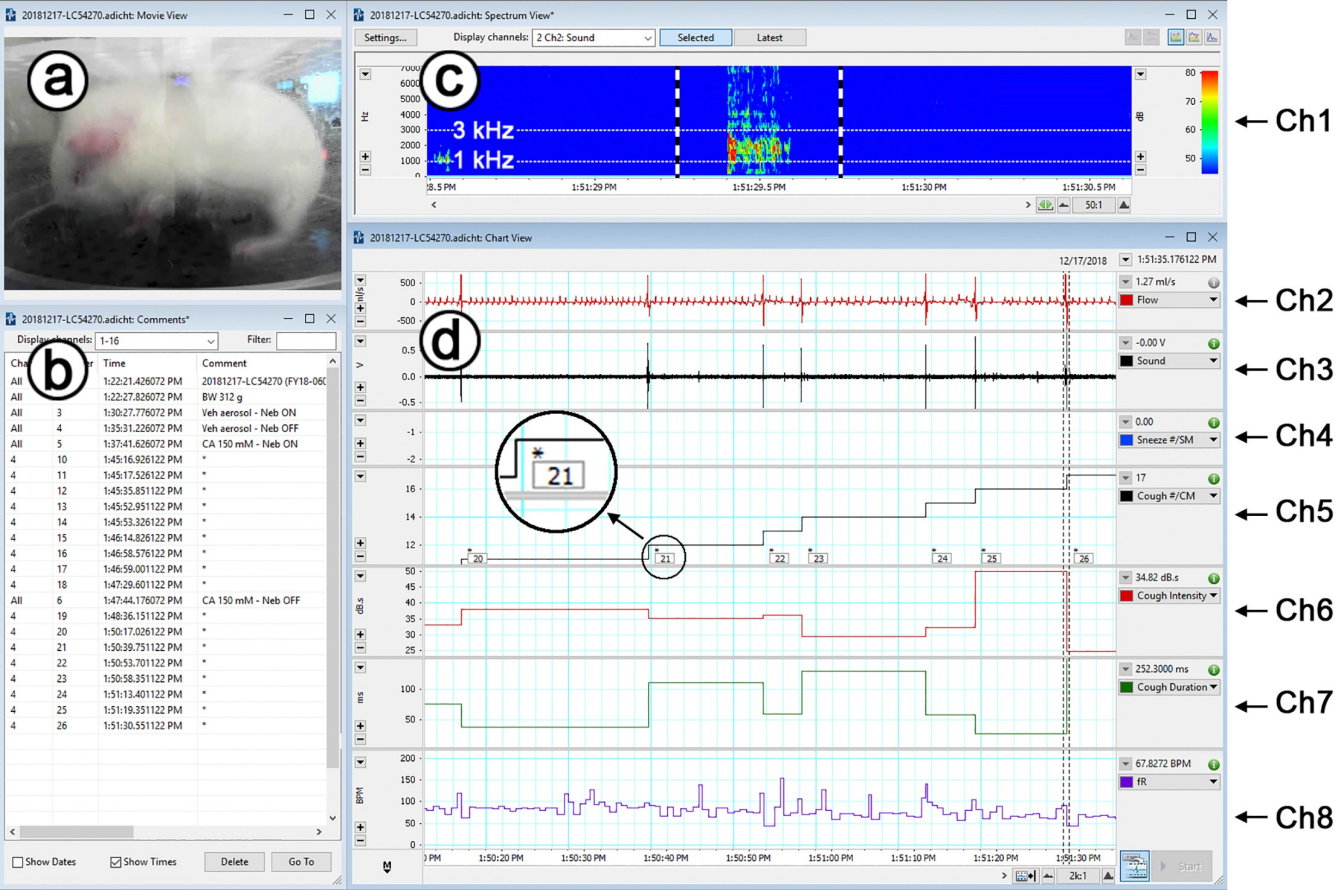
A screen shot showing our experimental recordings and analyses in real-time in a conscious guinea pig exposed to aerosolized CA. The symbols (a)—(d) represent four windows in the screen. On the left side, the real-time video monitoring of animal body posture and the list of comments are presented in a- and b-windows, respectively. The channels (Ch) on the right side from the top to bottom are spectrogram (Ch1 in c-window), respiratory flow, sound, accumulated sneeze count/sneeze marker (SM), accumulated cough count/cough marker (CM), cough sound integrated intensity (CSII), cough sound duration (CSD), and respiratory frequency (f_R_) (Ch2 through Ch8 in d-window). In real-time, SM and CM are manually marked by symbol “*”, while the accumulated sneeze and cough count, CSII, CSD, and f_R_ are automatically calculated and displayed on the screen. Note: the time-scale in c-window is set to 40-fold smaller than that in d-window for a better visual assessment of the cough-related spectrogram. Therefore, the cough spectrogram in c-window is corresponding to the last cough in d-window. The numbers with CM “*” in Ch5 as enlarged in the inset is the comment numbers but not the cough numbers. There are three columns in d-window: the scale and unit of the variable in each channel (left), the signals for each channel (middle), and the name and the value of each channel in real time (right). The lines in Ch5, 6 and 7 (the middle column) present the levels of cough numbers, intensity, and duration of individual coughs, while the values of the accumulated cough numbers, the cough intensity and duration of the last cough are displayed in Ch5, 6 and 7, respectively, of the right column.

Signals including animal body posture, respiratory flow, and cough sound were monitored and recorded, and cough numbers were counted in real-time. As shown in Fig 2 (a screen shot), the left top window (a) displays the video of animal body posture, and the left bottom window (b) shows the comment list for recording the general animal and event information. The spectrogram is presented in channel 1 (Ch1) of the top window (c) on the right side of the screen. It was generated by using fast discrete Fourier transform (FFT) from the sound waveforms (windowed with Hann window and transformed by 128 sample points with an overlap of 50%). The spectrogram was utilized here to assist in identification of the cough events visually. Previous studies have indicated that coughing has a peak PSD at ~1.5 kHz and sneezing has a peak PSD at 3.5–6.5 kHz [24, 25, 27, 28]. Thus, a typical cough was identified in real time by the following three criteria: (1) a transient and great change in the respiratory flow; (2) a cough sound with the peak power at ~1.5 kHz on the spectrogram; and (3) a cough-related body posture. Respiratory flow (Ch2) and cough sound (Ch3) are shown in the bottom window (d). Once a cough was encountered, the cough marker “*” (CM) was manually added by an observer in Ch5, in which the cough was also counted automatically in real time. Therefore, the accuracy of the cough numbers counted manually and automatically could be compared in real time. If the peak PSD occurred at 3.5–6.5 kHz, a sneeze would be manually marked by adding an “x” (SM) and automatically counted in Ch4 in real time. In addition, events of cough with wheeze and/or mucus were recognized according to the existence of a series of vertical lines and horizontal bands in the spectrogram and were manually noted in the comments. The cough sound integrated intensity (CSII), cough sound duration (CSD), and respiratory frequency variables are displayed in Ch6, Ch7, and Ch8 respectively. Real-time recordings of the CA-evoked coughs are shown in S1 Video, which provides dynamic cough analysis and compares coughs counted manually and automatically during recording.

#### The methodology of automatic cough (sneeze) number counting and determination of CSII and CSD in real time

**Fig 3:**
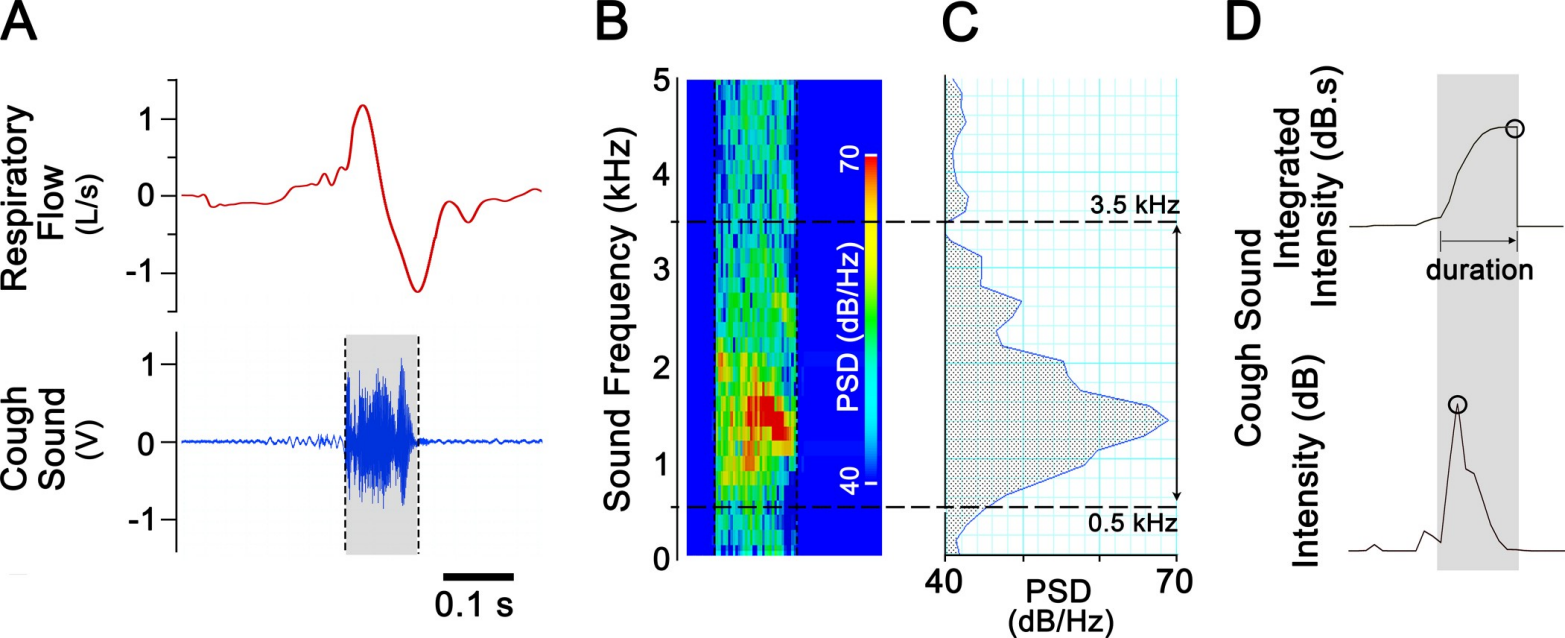
Acoustic analysis of a cough in response to inhalation of aerosolized CA in a conscious guinea pig. A: The respiratory flow (top, an expiration and its followed inspiration) and waveform of a cough sound (bottom, the shaded part). B: Spectrogram of the cough sound. C: The power spectrum density (PSD) distribution of this cough sound. The power is mainly located in frequencies from 0.5 to 3.5 kHz with a peak around 1.0 kHz. D: The cough sound integrated intensity (CSII, top) and the peak cough sound intensity (PCSI, bottom) are indicated by the cycles; the cough sound duration (CSD) is measured in the top panel.

The accumulated sneeze number, accumulated cough number, CSII, and CSD are exhibited in Ch4-7 (Fig 2). Cough numbers were automatically counted following three calculating steps. (1) A significant augmented respiratory flow characterized by a rapid expiration followed by a deep inspiration as a breathing cycle was used as the first criteria. The threshold used in this study was set at 400 ml/s. (2) The sound during this event was then used for determining a cough or sneeze. The total powers of the sound with the frequency ranges of 0.6–2.2 kHz and 3.5–6.5 kHz were summed. When either value was over 43 dB, the sound was recognized as a cough or sneeze. (3) If the sound with the frequency ranges of 0.6–2.2 kHz was greater than that with the frequency ranges of 3.5–6.5 kHz, this sound was determined to be a cough; otherwise, it was determined to be a sneeze. The two ranges of sound frequencies were selected in this study because the cough sound has been characterized by a peak PSD at ~1.5 kHz, and a sneeze sound has been characterized by a peak PSD at 3.5–6.5 kHz [24, 25, 27, 28]. Moreover, the peak PSD of a cough usually occurs at 0.6–2.2 kHz, as determined in our pilot experiment (also see Fig 3B and 3C for a typical example) and in our previous unpublished data. The cough or sneeze number was subsequently counted in an accumulating manner in Ch5 or Ch4. Subsequently, the CSII value and CSD (from the beginning to the end of the CSII signal) were calculated (Fig 3D, top), and the peak cough sound intensity (PCSI as shown in Fig 3D, bottom) was also analyzed (not shown in Fig 2).

#### Offline analysis of cough PSD

PSDs of cough sounds were generated using FFT with the same settings as for the spectrogram and were averaged for each cough (see Fig 3C). Due to the software limitations, the PSDs were analyzed from the recorded data but not in real time.

#### Ventilatory and other related variables

Respiratory frequency (f_R_), tidal volume (V_T_) and minute ventilation (V_E_) were calculated from the respiratory flow data in real-time. f_R_ was displayed in Ch8 (Fig 2), while V_T_ and V_E_ were in Ch9 and 10 (not shown). Other raw signals, such as chamber temperature, relative humidity, and oxygen concentration, were also recorded but not displayed due to the limited screen size.

### Experimental protocols

After completion of the adaptation, the guinea pigs were placed individually in the chamber. When the animal became stable for at least 5 min, aerosolized vehicle [n = 7, including 3 for saline (vehicle of CA) and 4 for 1% DMSO in saline (vehicle of PGE_2_)], CA (n = 7, 150 mM) [50], or PGE_2_ (n = 7, 0.43 mM) [42] was delivered into the chamber for 10 min and the animal stayed in the chamber for an additional 20 min after delivery. The dose of either aerosolized CA or PGE_2_ has been previously utilized to evoke Type I and II cough in conscious guinea pigs [42, 50]. Because neither saline nor saline+DMSO caused cough, their data were grouped together as the vehicle group in this study. Both CA and PGE_2_ were purchased from Sigma-Aldrich (St. Louis, MO). All variables described above were monitored and recorded continuously before and during aerosol delivery and 20 mins post-delivery [51]. After completion of the test, the animal was removed from the chamber and then euthanized with Euthasol (200 mg/kg, i.p.).

### Data acquisition and statistics

In this study, we characterized the following parameters: a) cough appearance (latency, number, rate, interval, and temporal distribution); b) cough acoustics (waveform, duration, PSD distribution, spectrogram, and intensity); and c) ventilation in response to aerosolized CA and PGE_2_. All data are presented as the means ± SE. Nonparametric statistical analysis (Mann-Whitney U test) was used for comparisons of the variables listed in Table 1; the peak cough intensity and the integrated cough intensity in response to CA and PGE_2_; and the PSDs at each frequency interval between the baseline noise and cough sound. Ventilatory variables (V_E_, V_T_ and f_R_) before CA or PGE_2_ exposure are expressed as absolute values, and ventilatory responses to CA or PGE_2_ exposure were measured every 5 min after initiation of the exposure and were normalized to their baselines (100%). Two-way ANOVA followed by Tukey’s test was used to compare the differences of the above variables between CA and PGE_2_ exposure. Significant differences were accepted when P < 0.05.

**Table 1:** Characteristics of coughs evoked by aerosol exposure to CA and PGE_2_.

Variables (mean ± SE)	Vehicle (N = 7)	Citric Acid (N = 7)	PGE_2_ (N = 7)
Total cough number (counts)	0.0 ± 0.0	32.7 ± 5.2 **	34.9 ± 8.5 **
Cough bout number (counts)	0.0 ± 0.0	0.0 ± 0.0	2.0 ± 0.6 **^‡^
Latency (min)	n/a	1.8 ± 0.3	3.8 ± 0.6 ^†^
Cough period (min)	n/a	17.3 ± 1.9	1.0 ± 0.6 ^‡^
Cough rate (coughs/min)	n/a	1.9 ± 0.3	53.1 ± 12.0 ^‡^
Average cough interval (s)	n/a	40.1 ± 10.6	0.8 ± 0.1 ^‡^
Average bout interval (min)	n/a	n/a	1.6 ± 0.4

** P < 0.01, vs. vehicle

^†^ P < 0.05

^‡^ P < 0.01

PGE_2_ vs. citric acid (CA). Note: the values of average bout interval were collected from 3 guinea pigs showing 2–5 bouts of coughing.

## Results

### CA and PGE_2_ exposure evoke coughs with different cough latency, rate, interval, and temporal distribution

**Fig 4:**
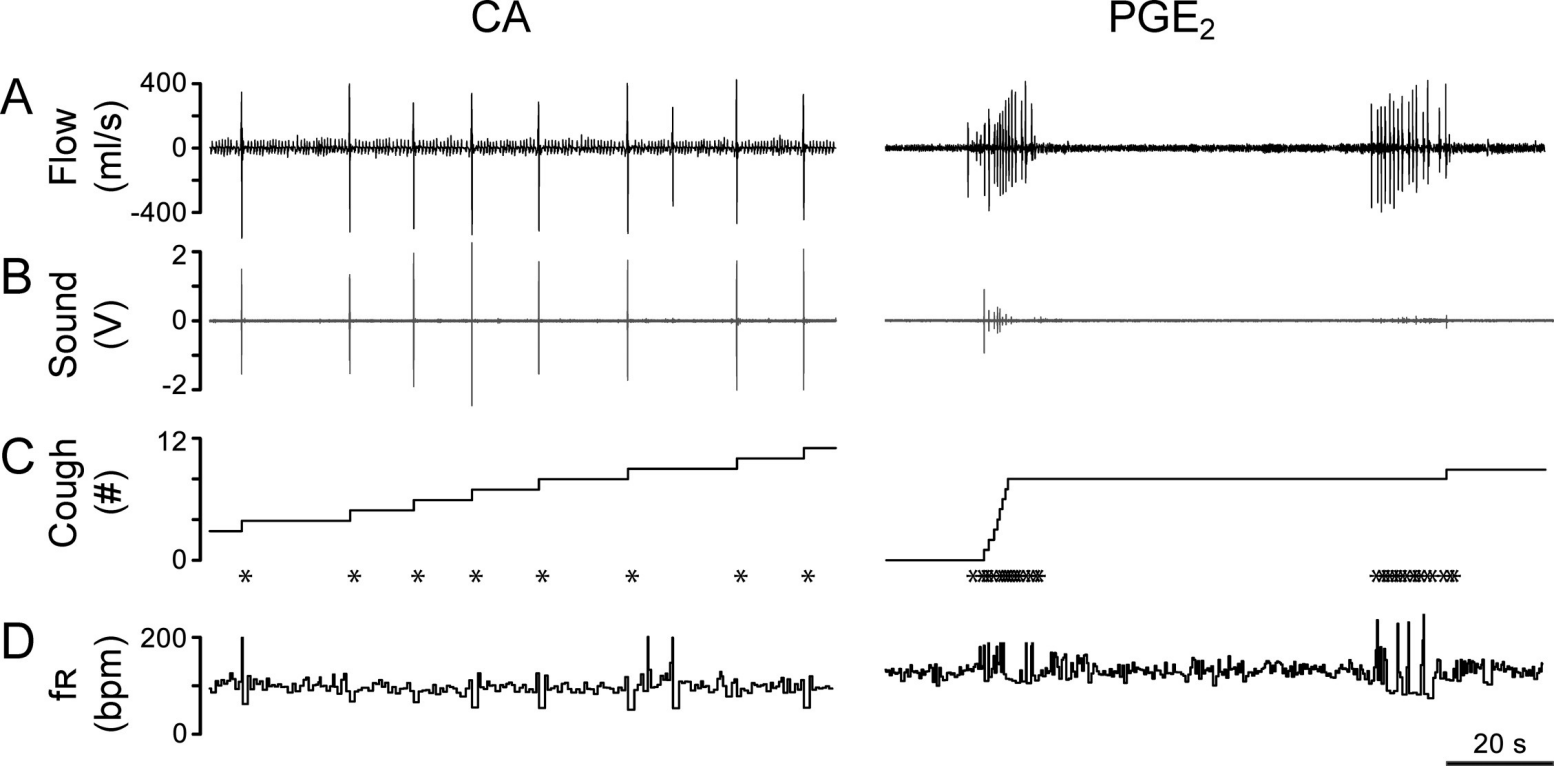
Comparison of the cough responses to CA or PGE_2_ exposures in two conscious guinea pigs. The traces from top to bottom are air flow (A), sound (B), cough number/cough marker (CM, C), and respiratory frequency (f_R_, D). Note: the coughs counted manually (with symbol “*”) and those counted automatically in C are perfectly matched for CA-evoked coughs but not for PGE_2_-evoked coughs because the sound signals in the latter are absent or much smaller than those in the former. Owing to the lack of cough response to vehicle, the relevant data were not shown here.

**Fig 5:**
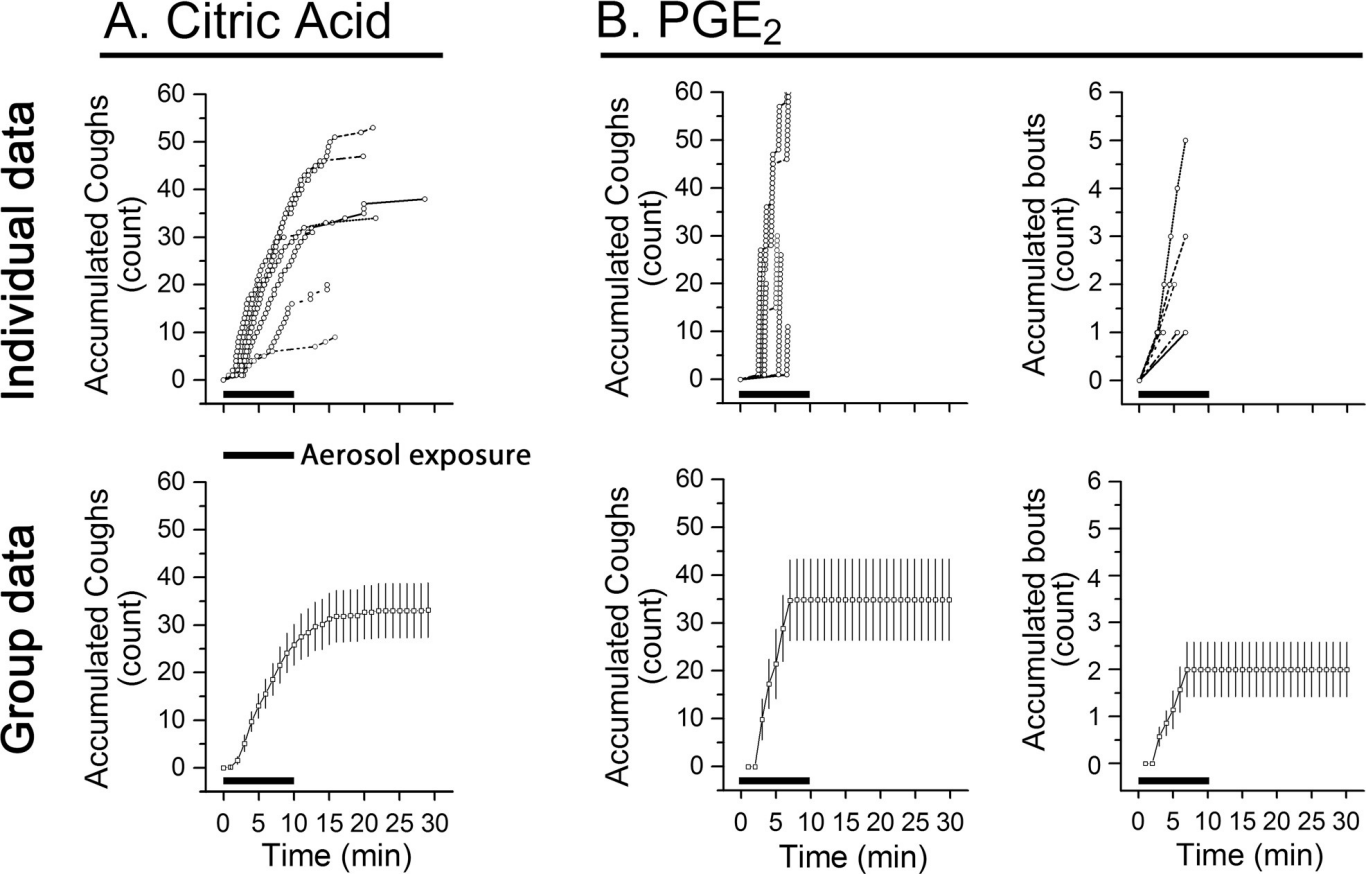
Distributions of cough responses to CA (left column for cough numbers) and PGE_2_ (middle and right columns for cough and bout numbers). The top and bottom in each column represent raw and group data. Horizontal bars = 10 min delivery of CA and PGE_2_ aerosol. N = 7 in each group; mean ± SE.

Inhalation of aerosolized CA (150 mM) or PGE_2_ (0.43 mM) for 10 min evoked the different types of coughs that were compared in Fig 4. The former evoked individual coughs, while the latter induced bout(s) of coughs. Both the amplitude of respiratory flow change and the raw cough sound were greater and louder in CA-evoked coughs than in PGE_2_-evoked coughs. Importantly, inhalation of CA and PGE_2_ produced similar cough numbers (32.6 vs. 34.9) with prominent differences in cough latency, rate, and interval (Table 1). Compared to PGE_2_, the latency of CA-induced cough was much shorter (1/2) with a strikingly longer period (17-fold) and lower cough rate. The average interval between two neighboring coughs was 50-fold shorter in PGE_2_- than in CA-evoked coughs (0.8 s vs. 40.1 s). In seven guinea pigs tested, PGE_2_ evoked one, two, three and five bouts of coughing in 4, 1, 1, and 1 animals, respectively. As illustrated in Fig 5, the accumulation of the evoked coughs was much faster in response to PGE_2_ than CA. Thus, the rate of the cough number accumulation was much higher in the PGE_2_-induced coughs than in the CA-evoked coughs. It should be noted that no sneezes were observed in the tested animals.

### The accuracy of automatic cough counting is much higher in response to CA than PGE_2_

In the 229 manually counted coughs in response to CA in 7 guinea pigs, 226 coughs (98.7%) were recognized automatically using our automated cough recording and analysis system. However, in the 244 coughs in response to PGE_2_ in an additional 7 guinea pigs, the accuracy of the automatic cough count was only ~35% (85 coughs recognized). As illustrated in Fig 4, cough numbers counted manually and automatically in response to CA were perfectly matched. However, this match was poor in the PGE_2_-evoked coughs due to either no or weak cough sound. No false count was found in the automatic counting.

### The two types of coughs possess distinct acoustic characteristics

**Fig 6:**
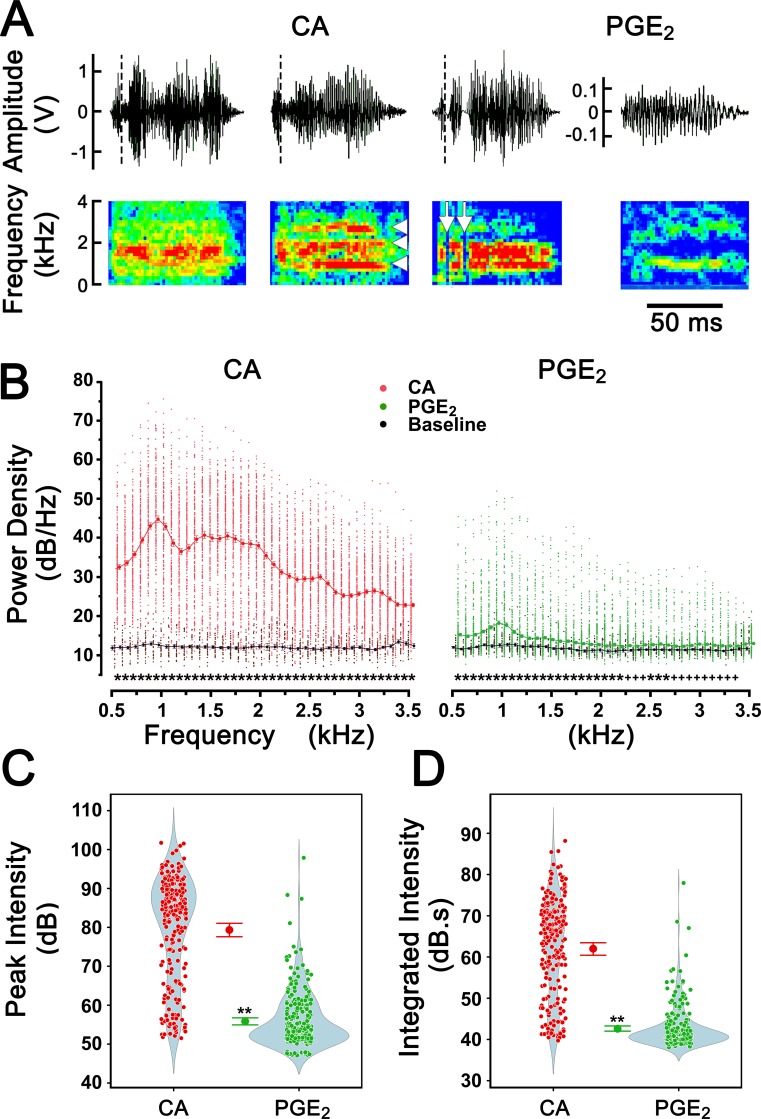
Acoustic analysis of coughs evoked by aerosolized CA and PGE_2_. Typical sound waveforms (top) and spectrograms (bottom) of the coughs evoked by CA and PGE_2_ are illustrated in A and B, respectively. A: At the top, the dashed lines separate the initial phases from the following phase in response to CA. The arrowheads in the middle panel of the spectrogram point to a series of horizontal bands, while the arrows in the right panel indicate the vertical lines. Note: The scale of the Y-axis at the top of B is different from that of A. B: Group data of sound power spectral densities (PSDs) in which the spots are the individual values of the PSDs (229 and 244 coughs in the CA and PGE_2_ groups respectively), and the lines with markers represent the averaged values and standard errors. The baseline values of power density (5 data points from each animal) were generated from the sound recordings before the first 5 coughs provoked by CA or PGE_2_ exposure. Compared to the baseline noise at a given audio frequency, PSDs in response to CA (0.5 to 3.5 kHz, P < 0.01) and PGE_2_ (0.5 to 2.75 kHz, P < 0.01; and 2.8 to 3.3 kHz, P < 0.05) are significantly higher. Both CA- and PGE2-evoked coughs have peak PSDs located over a range of 0.6 to 2.2 kHz, with the topmost value at approximately 1.0 kHz, but the PSDs at the corresponding frequencies in the PGE2-evoked coughs are much lower than those in CA-evoked coughs. C and D: Comparisons of the peak sound intensity and the integrated intensity between CA and PGE_2_ groups, respectively. The scattered dots are individual data and the dots with standard error bars are the mean values. The shaded areas indicate the distributions of individual data. All group data from 7 guinea pigs from the CA and PGE_2_ groups are presented as the mean ± SE. ** P < 0.01, PGE_2_ vs. CA.

We analyzed the cough sound waveforms and spectrograms in response to CA and PGE_2_. The CSD values in response to CA and PGE_2_ were much shorter (86 ± 15 ms and 78 ± 14 ms) than cough sounds observed in humans (210–355 ms) [31]. Cough sound waveforms in response to CA contained two phases, an initial brief phase (~10 ms) followed by a much longer phase (~90 ms) whose shape was largely varied in each cough, while those in response to PGE_2_ usually lacked the initial phase (Fig 6A, top). The ratio of the duration of phase-1: phase-2 in response to CA in guinea pigs (10 ms/90 ms = 11%) is shorter than that observed in humans (40 ms/160 ms = 25%) [31]. Interestingly, a series of vertical lines (3%) or horizontal bands (31%) that indicate cough with mucus and wheezes in humans was also observed in the cough responses to CA but not to PGE_2_ (Fig 6A, bottom). We further analyzed and compared PSD distribution and cough sound intensity in the CA and PGE_2_ groups. There were several significant findings from the data depicted in Fig 6B–6D. (i) The PSDs were completely discernible from the baseline noise in the CA groups in the spectral range from 0.5–3.5 kHz and in the PGE_2_ group in the spectral range from 0.5–3.35 kHz. (ii) In both groups, the PSD peaked at ~1.0 kHz. (iii) The PSD distributions of the two types of coughs were similar, and the amplitudes of averaged PSDs were more than 100-fold higher in the CA- than PGE_2_-evoked coughs around the peak values. (iv) CSII and PCSI were ~125-fold higher in the CA- than PGE_2_-provoked coughs.

### CA but not PGE_2_ exposure induces hyperventilation

**Fig 7:**
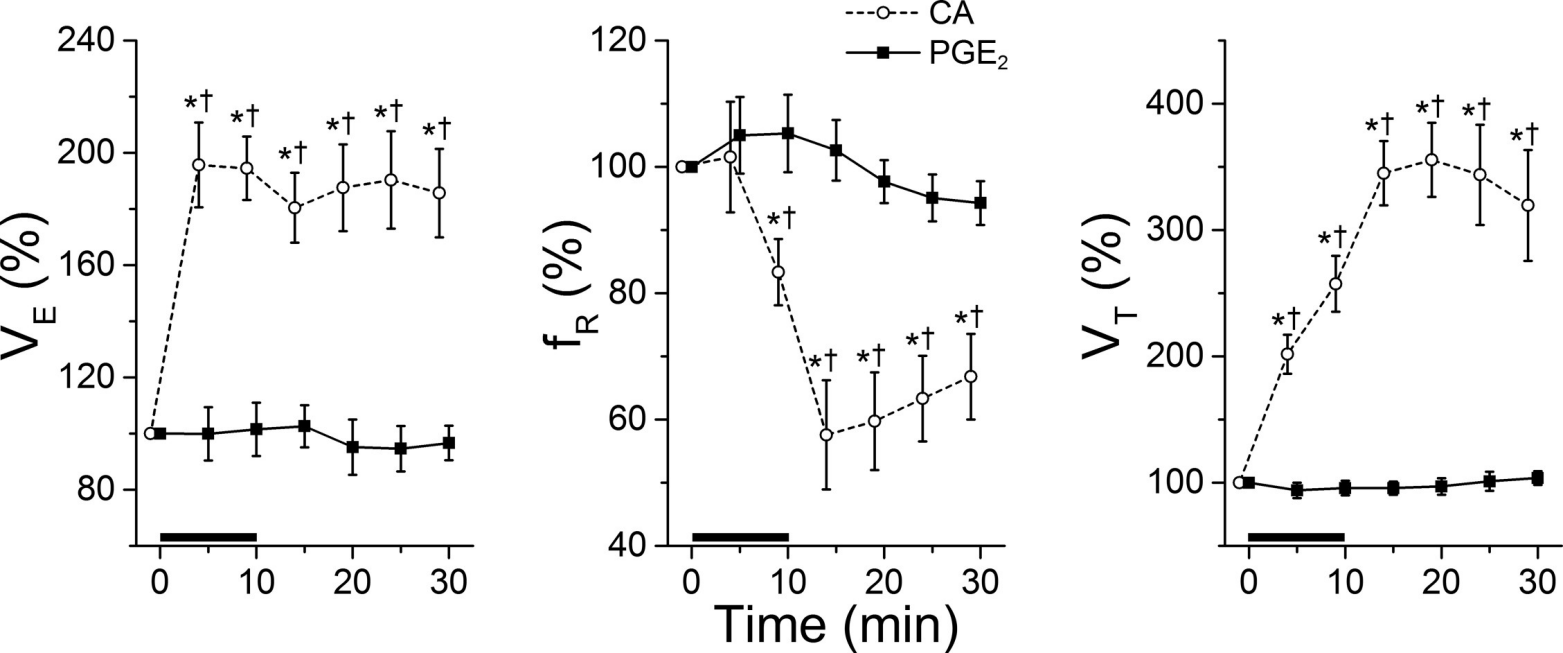
Ventilatory responses to inhalation of aerosolized CA and PGE_2_. The responses of minute ventilation (V_E_), respiratory frequency (f_R_), and tidal volume (V_T_) to CA and PGE_2_ exposure are presented in the left, middle, and right panels, respectively. N = 7 in each group; mean ± SE. * P < 0.01, compared to baseline values (“100%”) and † P < 0.01 compared to the PGE_2_-induced responses.

The baseline V_E_, f_R_, and V_T_ (before CA or PGE_2_ exposure) were not different between the CA and PGE_2_ groups. The values are 292 ± 21 vs. 298 ± 25 (V_E_, ml/min), 112 ± 5 vs. 118 ± 4 (f_R_, breaths/min), and 2.7 ± 0.2 vs. 2.6 ± 0.3 (V_T_, ml). The ventilatory responses to CA and PGE_2_ exposure are illustrated in Fig 7. CA exposure always induced immediate hyperventilation with a latency of approximately 1.5 min that reached plateau at 5 min after beginning of the exposure and this change persisted during 20 mins post-delivery. The V_E_ change resulted from a reduction of f_R_ by ~40% and an increase in V_T_ by ~3.5-fold. In sharp contrast, PGE_2_ exposure did not significantly alter V_E_, f_R_, and V_T._ In addition, vehicle exposure did not alter ventilation.

## Discussion

A typical cough response in animals has been characterized by the simultaneous appearance of remarkable and unique changes in the respiratory flow, sound, and body posture (three key signals) [24, 25, 27, 28]. However, the disadvantages of the previous recording system are obvious in these studies. First, though respiratory flow and sound were simultaneously recorded, the animal body posture was watched by observer(s) during the experiment without video recording. Because the changes of these variables are transient, matching them in a real-time manner is difficult during the experiment, which could lead to miscounting of cough numbers. Second, discrimination of cough from sneeze was manually counted by playback of the recordings (offline) in which the synchronized signals of the body posture were lacking, which markedly diminishes the accuracy and efficiency of identifying a cough. Third, cough sound duration and intensity synchronized with respiratory signals were not previously quantitatively analyzed in animal cough models, while it has been extensively applied in clinical settings [4–13]. Fourth, analysis of sound waveforms and spectrograms enables discrimination of productive and non-productive cough sounds in humans [31, 32]; however, such analysis has not been investigated in animals. Our recording/analysis system is novel because all the three key signals are simultaneously monitored and recorded, and importantly, there is a very high accuracy in automatically counting the coughs with louder sound (accuracy = 99% in CA-evoked coughs (see Fig 2 and S1 Video). Compared to CA, the accuracy of automatic counting coughs in response to PGE_2_ is much lower (~35%) owing to no or weak cough sound. Airflow changes during the PGE_2_-evoked cough are uniquely characterized by appearance of a series of large expiration in a bout manner (Fig 4). Thus, using this airflow characteristic may be another way to define Type II cough. Importantly, our recording/analysis system is capable of analyzing the acoustic signals (waveform, spectrogram, CSII, and CSD in real-time; and PSD distribution and PCSI offline). The acoustic signals of cough can be applied to easily distinguish cough from other expiratory activities that lack cough sound. An expiration reflex, sigh, and/or sniffing also contain a relatively large expiration [52, 53]. Though these expiratory activities exist in conscious guinea pigs, they lack the cough sound [52]. Therefore, our recording/analysis system provides the objectivity, accuracy and efficiency of counting cough numbers, offers the ability to differentiate cough patterns, and adds the cough sound intensity to assist in the determination of cough intensity.

Type I and II coughs evoked by aerosolized CA/capsaicin and PGE_2_ have been defined in humans and conscious guinea pigs [36–42]. However, the characteristics of the two types of coughs have not been investigated systematically. By using our recording and analysis system, we comprehensively and quantitatively differentiated the cough appearance (latency, number, rate, interval, and distribution) and cough acoustics (waveform, spectrogram, CSII, CSD, PCSI and PSD distribution) between the two types of coughs in guinea pigs. The prominent differences between the two types of coughs can be summarized as follows. i) Although the cough numbers induced by inhalation of 150 mM CA and 0.43 mM PGE_2_ for 10 min are similar (32.7 vs. 34.9), CA evokes individual loud coughs but PGE_2_ provokes bout(s) of coughs with low or absent sound, consistent with the previous reports [36–42]. ii) Compared to PGE_2,_ CA-induced coughs have shorter latency (1.8 s vs. 3.8 s) and a much lower rate (2/min vs. 53/min). iii) Both types of coughs possess the same PSD peaked at ~1.0 kHz; however, the PSD amplitude, but not the PSD distribution, in the CA-evoked coughs strikingly differs from that in the PGE_2_-evoked coughs (Fig 6C). iv) Cough sound durations were similar in response to CA and PGE_2_ (86 ms vs. 78 ms), and the cough sound waveforms contain two phases in response to CA but usually only one phase in response to PGE_2_ (top of Fig 6A and 6B). v) The vertical lines and horizontal bands in the spectrograms that reflect cough with mucus and wheeze, respectively, as reported in humans [31–35] were observed in a few cough responses to CA (bottom of Fig 6A). vi) Both the peak and integrated intensity of cough sound are ~100-fold stronger in the CA- than the PGE_2_-induced coughs (Fig 6C and 6D), indicating that cough sound intensity is a sensitive parameter to detect cough intensity. Importantly, our data, so far as we know, first to reveal the cough sound spectrum elicited by PGE_2_, to compare the PSDs between the two types of coughs and to demonstrate the cough sound intensity as an another index applicable for quantitative determination of cough intensity. Furthermore, the high sensitivity of CSII observed in this study offers the ability to unmask the antitussive impact of a putative drug in some cases in which the antitussive effect is mainly on the cough intensity rather than the cough number.

The cough sound in clinical settings is a very important symptom of over 100 diseases [54]. The cough sound (timbre) has been applied to not only determine cough intensity but also to point to the clinical diagnosis, such as COPD, asthma, chronic bronchitis, and tracheobronchial collapse [12, 14, 15]. Recently, through examining the waveforms and spectrograms (frequency content), investigators interpret distinct coughs with mucus and/or wheeze or pulmonary diseases, such as idiopathic pulmonary fibrosis, COPD, asthma and laryngitis [31–35]. As reported, there are three phases in a cough sound waveform in patients, and coughs with mucus and wheezes are distinguished by the unique existence of vertical lines and horizontal bands in the spectrograms [31–33]. The diagnostic value of different types of cough has been confirmed to recognize the specific features of whooping cough, bronchiolitis, croup, and cough associated with tracheoesophageal fistula [55, 56]. In fact, the presence of different types of cough (i.e., dry, moist, productive, brassy, hoarse, wheezy, barking, etc.) indicates the clinical value of these sound properties. Therefore, the development of ambulatory cough recording/analysis devices has been considered as an active and competitive research field [10, 11, 57]. With respect to animal cough models, the monitoring and analysis of cough sounds have been utilized in infected weaned calves [30] and pigs [28, 29], and certain types of cough sounds have been correlated to their corresponding diseases [28, 29]. For example, pigs infected with *Pasteurella multocida* or *Actinobacillus pleuropneumoniae* had a significantly reduced peak cough PSD, and the CSD was shortened by 30% compared with healthy pigs [28, 29]. Our recording/analysis system possesses the unique ability to simultaneously monitor, record, and analyze cough and cough sound acoustics in a real-time manner. This new system may benefit discrimination of cough patterns in animal models with pulmonary diseases in future studies.

Sneezes in response to capsaicin aerosol for 10 min were observed in an early study, but their occurrence in which group of guinea pigs (control, primary ovalbumin-sensitized, and multiple ovalbumin-sensitized) was, unfortunately, not clarified [25]. It is generally accepted that only mechanical stimulation or a wide variety of chemical irritants locally applied to the nasal mucosa can cause sneezing (for a review, see [58]). For example, bilateral local stimulation by instillation of capsaicin, ovalbumin, substance P, or histamine into the nasal cavity induces sneezes in conscious guinea pigs sensitized by ovalbumin [59–61]. Inhalation of CA and PGE_2_ aerosol fails to induce sneeze in our guinea pigs without ovalbumin sensitization, which is in agreement with the reports mentioned above. Further studies are essential to validate the ability of our recording/analysis system to automatically define and count sneezes in animals, such the ovalbumin-sensitized guinea pig with chemical irritants locally applied to the nasal mucosa.

In addition to cough, our results further found different respiratory responses to CA and PGE_2_. CA exposure induces an immediate and remarkable elevation in V_E_, as previously reported in conscious lambs [43], while PGE_2_ exposure fails to significantly alter V_E_, similar to observations in dogs [44]. The mechanisms underlying the difference between the two types of cough/ventilatory responses evoked by CA and PGE_2_ remain unknown. Cough reflex is triggered by vagal pulmonary sensory afferent nerves including bronchopulmonary C-fibers (PCFs) and Aδ-fibers (irritant receptors)(for a review, see [58]). The CA-induced coughs are mainly mediated by acting on TRPV1 [15, 27, 45–49] of PCFs and also by the vagal pulmonary Aδ-fibers [62]. On the other hand, activation of vagal sensory afferents’ EP_3_ is responsible for the PGE_2_-evoked coughs [42]; however, it remains unclear which type(s) of vagal sensory fibers is/are involved. Thus, one explanation for the different types of coughs is due to the differences in the types and/or the population of vagal sensory fibers/receptors activated by CA and PGE_2_. Furthermore, it is needed to determine whether the sensory afferents expressing TRPV1 and EP_3_ have different neural loops in the brainstem. Another possible explanation is the different deposition locations between the CA and PGE_2_ aerosol because the coughs are largely varied by the different stimulating sites within the airways [52, 63, 64]. Aerosol deposition in the airways is mainly affected by the droplet sizes of the aerosolized solutions. For example, the cough response to the same dose of capsaicin is much higher in humans exposed to a large droplet size (mass median diameter = 5.2 μm) than those exposed to a small one (mass median diameter = 3.2 μm) [65]. In the present study, CA and PGE_2_ were dissolved in saline without and with 1% DMSO respectively. Therefore, further clarification of the differences in the aerosol droplet size and airway deposition location between CA and PGE_2_ aerosol is necessary to determine the contribution of these differences to the two types of coughs. Actually, the pattern of respiratory frequency in response to PCF activation reportedly depends on the stimulating intensity. For example, stimulation of PCFs via right atrial injection of capsaicin and phenylbiguanide at a low dose (slow infusion) evokes rapid ventilation, but at a high dose (bolus injection) induces apnea [66–70]. One may ask whether the different CA- and PGE_2_-evoked patterns of coughs and ventilatory response are switchable by manipulation of the stimulating intensity. Studies in the conscious guinea pig have shown that the cough numbers (Type I) are positively correlated to CA doses (50, 100, 200, 400, and 800 mM) delivered into the larynx and plateaued at 400 mM [52]. Moreover, the bouts of coughs in response to aerosolized PGE_2_ at 30, 100, and 300 μg/ml are zero, 3.3, and 12.6 respectively [42]. These data support that the nature of Type I and Type II cough is unchangeable and independent of CA and PGE_2_ doses; however, it remains unknown whether the hyperventilation elicited by CA and the lack of ventilatory response to PGE_2_ are dose-independent.

## Perspective

Cough is the most common symptom in clinical settings (accounting for one third of pulmonologist consultations) [3, 71, 72]. Changes of the cough sound may indicate the effectiveness of therapy or the progress of disease. Animal cough models have been extensively used to develop new antitussive drugs. This study establishes a novel system by which all signals including cough-relayed body posture, respiratory flow, cough appearance and acoustics are simultaneously recorded and analyzed in real-time. This system provides the objectivity and accuracy of counting cough numbers and gains the ability for differentiating cough pattern and determining cough intensity. Further study is required to validate the applicability of our system to discriminate cough patterns in animals with pulmonary diseases, such as idiopathic pulmonary fibrosis, COPD, or asthma.

## Supporting information

S1 Video A video showing our experimental recordings and analyses in real-time in a conscious guinea pig exposed to aerosolized CA. The left side of the screen presents the animal body posture and comments window, while the right side shows spectrogram, respiratory flow, sound, sneeze number/sneeze marker, cough number/cough marker, cough sound integrated intensity (CSII), cough sound duration (CSD), and respiratory frequency (f_R_) from the top to bottom, similar to those presented in Fig 2. This video provides dynamic cough analysis and compares coughs counted manually and automatically in real-time.
(MP4)
